# Hyperprogression of a spindle‐cell squamous carcinoma of the esophagus after esophagectomy

**DOI:** 10.1111/1759-7714.14526

**Published:** 2022-06-06

**Authors:** Yanli Ji, Mei Yang, Chengwu Liu, Wenping Wang

**Affiliations:** ^1^ Department of Thoracic Surgery West China Hospital, Sichuan University/West China School of Nursing, Sichuan University Chengdu China; ^2^ Department of Thoracic Surgery West China Hospital, Sichuan University Chengdu China

A 49‐year‐old man with esophageal spindle‐cell squamous carcinoma suffered tumor hyperprogression and leukemoid reaction after esophagectomy. He had presented with progressive dysphagia when he came to our clinic on an outpatient basis. Chest computed tomography (CT) scans indicated a 4 × 2.5 cm mass in the lower part of the esophagus (Figure 1(a)). Gastroscopy identified a neoplasm which was located in the esophagus 35–39 cm from the incisors, and squamous carcinoma was confirmed following initial biopsy and histological diagnosis. Preoperative tumor work‐up examinations did not reveal local or distant metastases. He subsequently underwent a radical endoscopic McKeown esophagectomy. The pathological diagnosis confirmed a spindle‐cell squamous carcinoma (pT3N2M0). Esophageal sarcomatoid squamous cell carcinoma is known as a rare esophageal malignant tumor.^1^ Our patient recovered well during the first week after the operation and began to feed himself on postoperative day 7. However, he subsequently developed chylothorax and persistent fever (38.5 to 39°C) immediately after taking food, with no sign of anastomotic leakage and no clinical evidence of infection. Meanwhile, he experienced progressive leukocytosis and a sharp increase in white blood cell count (Figure 1(b)). Contrast enhanced chest CT scan on day 28 after surgery demonstrated extended soft tissues in the mediastinum, massive pleural effusion in the left thoracic cavity, and numerous pleural nodules (Figure 1(c)). Thoracoscopic examination showed pleural dissemination of nodules in the left thoracic cavity (Figure 1(d)), and nodule biopsies confirmed metastases of spindle‐cell squamous carcinoma. He gradually developed cachexy, respiratory failure and did not receive adjuvant therapy because of his poor physical status. Although strong supportive therapies were administered, he suffered progressive disease deterioration and died from cardiorespiratory failure 33 days after the surgery.

**FIGURE 1:**
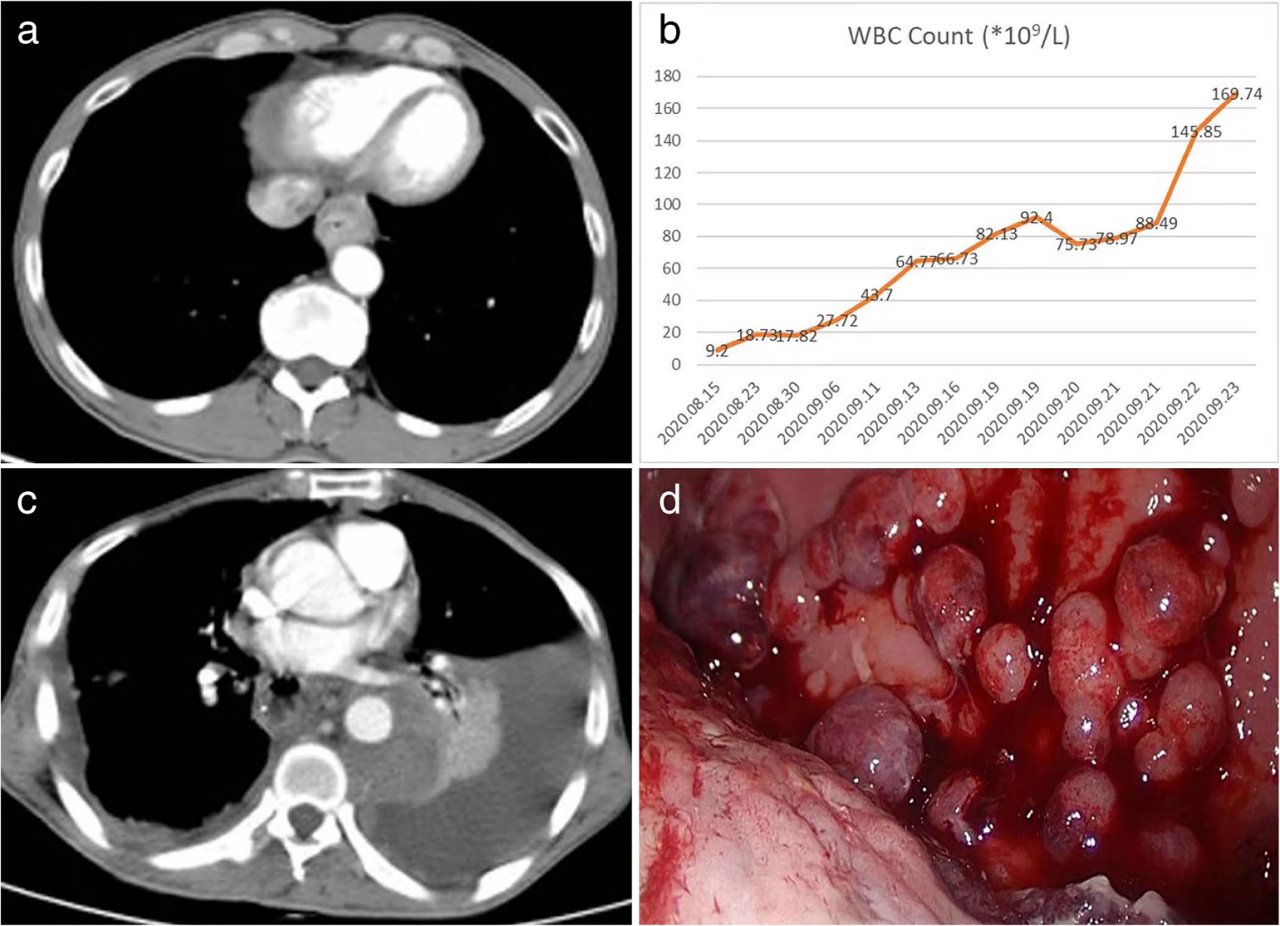
(a) Chest computed tomography (CT) revealed a mass in the lower part of the esophagus. (b) Perioperative tendency of the WBC. (c) Chest CT scans 28 days after the surgery revealed soft tissues around the descending aorta, massive pleural effusion in the left thoracic cavity, and numerous pleural nodules. (d) Thoracoscopy revealed pleural dissemination with nodules in the left thoracic cavity

## CONFLICT OF INTEREST

The authors declare no competing interests.
